# Etiologies of severe acute respiratory infection (SARI) and misdiagnosis of influenza in Indonesia, 2013‐2016

**DOI:** 10.1111/irv.12781

**Published:** 2020-07-14

**Authors:** Abu Tholib Aman, Tri Wibawa, Herman Kosasih, Rizka Humardewayanti Asdie, Ida Safitri, Umi Solekhah Intansari, Yuli Mawarti, Pratiwi Sudarmono, Mansyur Arif, Dwiyanti Puspitasari, Bachti Alisjahbana, Ketut Tuti Merati Parwati, Muhammad Hussein Gasem, Dewi Lokida, Nurhayati Lukman, Teguh Sarry Hartono, Yan Mardian, C Jason Liang, Sophia Siddiqui, Muhammad Karyana, Chuen‐Yen Lau

**Affiliations:** ^1^ Department of Microbiology, Faculty of Medicine, Public Health, and Nursing Universitas Gadjah Mada / Dr. Sardjito Hospital Yogyakarta Indonesia; ^2^ Indonesia Research Partnership on Infectious Diseases (INA‐RESPOND) Jakarta Indonesia; ^3^ Department of Internal Medicine, Faculty of Medicine, Public Health, and Nursing Universitas Gadjah Mada / Dr. Sardjito Hospital Yogyakarta Indonesia; ^4^ Department of Pediatric, Faculty of Medicine, Public Health, and Nursing Universitas Gadjah Mada / Dr. Sardjito Hospital Yogyakarta Indonesia; ^5^ Department of Clinical Pathology, Faculty of Medicine, Public Health, and Nursing Universitas Gadjah Mada / Dr. Sardjito Hospital Yogyakarta Indonesia; ^6^ Cipto Mangunkusumo Hospital, Faculty of Medicine Universitas Indonesia Jakarta Indonesia; ^7^ Faculty of Medicine Universitas Hasanudin / Dr. Wahidin Sudirohusodo Hospital Makassar Indonesia; ^8^ Dr. Soetomo Academic General Hospital, Faculty of Medicine Universitas Airlangga Surabaya Indonesia; ^9^ Faculty of Medicine Universitas Padjadjaran / Dr. Hasan Sadikin Hospital Sumedang Indonesia; ^10^ Medical Faculty Udayana University and Sanglah General Hospital Denpasar Indonesia; ^11^ Dr. Kariadi Hospital / Diponegoro University Semarang Indonesia; ^12^ Tangerang District Hospital Tangerang Indonesia; ^13^ Sulianti Saroso Hospital Jakarta Indonesia; ^14^ National Institute of Allergy and Infectious Diseases (NIAID) National Institutes of Health Bethesda MD USA; ^15^ National Institute of Health Research and Development (NIHRD), Ministry of Health Jakarta Indonesia

**Keywords:** diagnostic accuracy, etiology, Indonesia, influenza, severe acute respiratory infection

## Abstract

**Background:**

Severe acute respiratory infection (SARI) accounts for a large burden of illness in Indonesia. However, epidemiology of SARI in tertiary hospitals in Indonesia is unknown. This study sought to assess the burden, clinical characteristics, and etiologies of SARI and concordance of clinical diagnosis with confirmed etiology.

**Methods:**

Data and samples were collected from subjects presenting with SARI as part of the acute febrile Illness requiring hospitalization study (AFIRE). In tertiary hospitals, clinical diagnosis was ascertained from chart review. Samples were analyzed to determine the “true” etiology of SARI at hospitals and Indonesia Research Partnership on Infectious Diseases (INA‐RESPOND) laboratory. Distribution and characteristics of SARI by true etiology and accuracy of clinical diagnosis were assessed.

**Results:**

Four hundred and twenty of 1464 AFIRE subjects presented with SARI; etiology was identified in 242 (57.6%), including 121 (28.8%) viruses and bacteria associated with systemic infections, 70 (16.7%) respiratory bacteria and viruses other than influenza virus, and 51 (12.1%) influenza virus cases. None of these influenza patients were accurately diagnosed as having influenza during hospitalization.

**Conclusions:**

Influenza was misdiagnosed among all patients presenting with SARI to Indonesian tertiary hospitals in the AFIRE study. Diagnostic approaches and empiric management should be guided by known epidemiology. Public health strategies to address the high burden of influenza should include broad implementation of SARI screening, vaccination programs, clinician education and awareness campaigns, improved diagnostic capacity, and support for effective point‐of‐care tests.

## 1 INTRODUCTION

Severe acute respiratory infection (SARI) was defined in 2011 for purposes of global surveillance. The 2011 definition harmonized heterogeneous definitions used by three WHO regions, thus facilitating comparisons. The revisions include one definition for all age groups to simplify implementation, dropping “shortness of breath” and “breathing difficulty,” adding “history of fever” and increasing the onset of symptoms to 10 days. SARI is now defined as an acute respiratory illness with a history of fever or measured fever of ≥38°C and cough, with onset within the past 10 days and requiring hospitalization.^1^ This case definition enables monitoring of severe influenza‐related diseases and assessment of burden.

Severe acute respiratory infection criteria are not specific for influenza. A number of respiratory viruses other than influenza and bacterial etiologies in patients that met SARI criteria have been reported from previous studies.^2^, ^3^, ^4^ Several viruses and bacteria that cause systemic diseases such as dengue virus,^5^, ^6^ chikungunya virus,^7^, ^8^ *Salmonella spp*.,^9^ *Leptospira spp*.,^10^ and *Rickettsia typhi* ^11^ may present with a respiratory illness and fulfill the SARI criteria. A few studies have evaluated the sensitivity and specificity of SARI criteria for influenza detection. Sensitivity and specificity range from 37% to 84% and 23% to 78%, respectively.^12^, ^13^, ^14^ SARI criteria may also be used for pneumonia surveillance.^13^

Severe acute respiratory infection and influenza‐like illness (ILI) surveillance have been conducted in Indonesia since 1999 at several hospitals and primary health centers. These studies reported that the proportion of influenza cases varied from 14% to 20% of all enrolled subjects.^15^, ^16^, ^17^, ^18^ As the aims of these studies were to confirm influenza infections, other causes of respiratory infections or “systemic” viral or bacterial infections were not analyzed.

Since bacterial, influenza, and non‐influenza viral respiratory infections are prevalent in Indonesia, there is a need to evaluate strategies for respiratory pathogen surveillance in the region. Our study aimed to identify pathogens associated with SARI in Indonesia, to compare these with hospital assessed etiology, and to describe their demographic and clinical characteristics. In addition, we determined performance characteristics of SARI criteria for identification of influenza virus.

## 2 METHODS

### 2.1 Participants

Patients fulfilling the criteria for SARI^1^ were identified from an observational cohort study of patients hospitalized with acute febrile illness requiring hospitalization (AFIRE) conducted in Indonesia from 2013 to 2016.^19^ The AFIRE study recruited patients presenting to 8 tertiary hospitals for evaluation of acute fever, at least 1 year old, hospitalized within the past 24 hours, and not hospitalized within the past 3 months. These hospitals were top referral provincial hospitals representing 7 major cities (Bandung, Denpasar, Jakarta, Makassar, Semarang, Surabaya, and Yogyakarta) in three populous islands. Patients were eligible for participation if they or a legal guardian/representative provided written consent following an explanation of the study objectives and procedures in Indonesian. Assent was obtained from children ≥13 years old or who were old enough to understand the proposed research.

Informed consent or assent was obtained prior to collection of clinical data, laboratory data, and specimens. Blood was collected from all subjects at enrollment, 14‐28 days, and 3 months after enrollment. Other biological specimens such as nasopharyngeal swabs, sputum, urine, or feces were collected per the attending physician. Records were reviewed for demographic and clinical information, including clinical diagnoses made during hospitalization. The “true” diagnosis was determined based on hospital and/or Indonesia Research Partnership on Infectious Disease (INA‐RESPOND) laboratory testing in conjunction with available clinical information.

Severe acute respiratory infection was assessed per the WHO definition requiring (a) an acute respiratory illness (ARI), (b) history of fever or measured fever of ≥38°C, (c) cough, (d) onset within the past 10 days, and (e) requiring hospitalization.^20^ As no standard definition of ARI was available, we defined ARI as any sign or symptom related to the respiratory tract, including coryza, nasal congestion, sore throat, hemoptysis, and dyspnea. Presence of cough was determined by chart review. For patients who had pneumonia, it was assumed that cough was present even if not recorded. The requirements of fever, onset <10 days, and requiring hospitalization were already met by participants through AFIRE inclusion criteria. Ethical clearance was obtained from the institutional review board of the National Institute of Health Research and Development, Ministry of Health of Indonesia (number: KE 01.05/EC/407/ 2012).

### 2.2 Diagnostic testing

**Figure 1:**
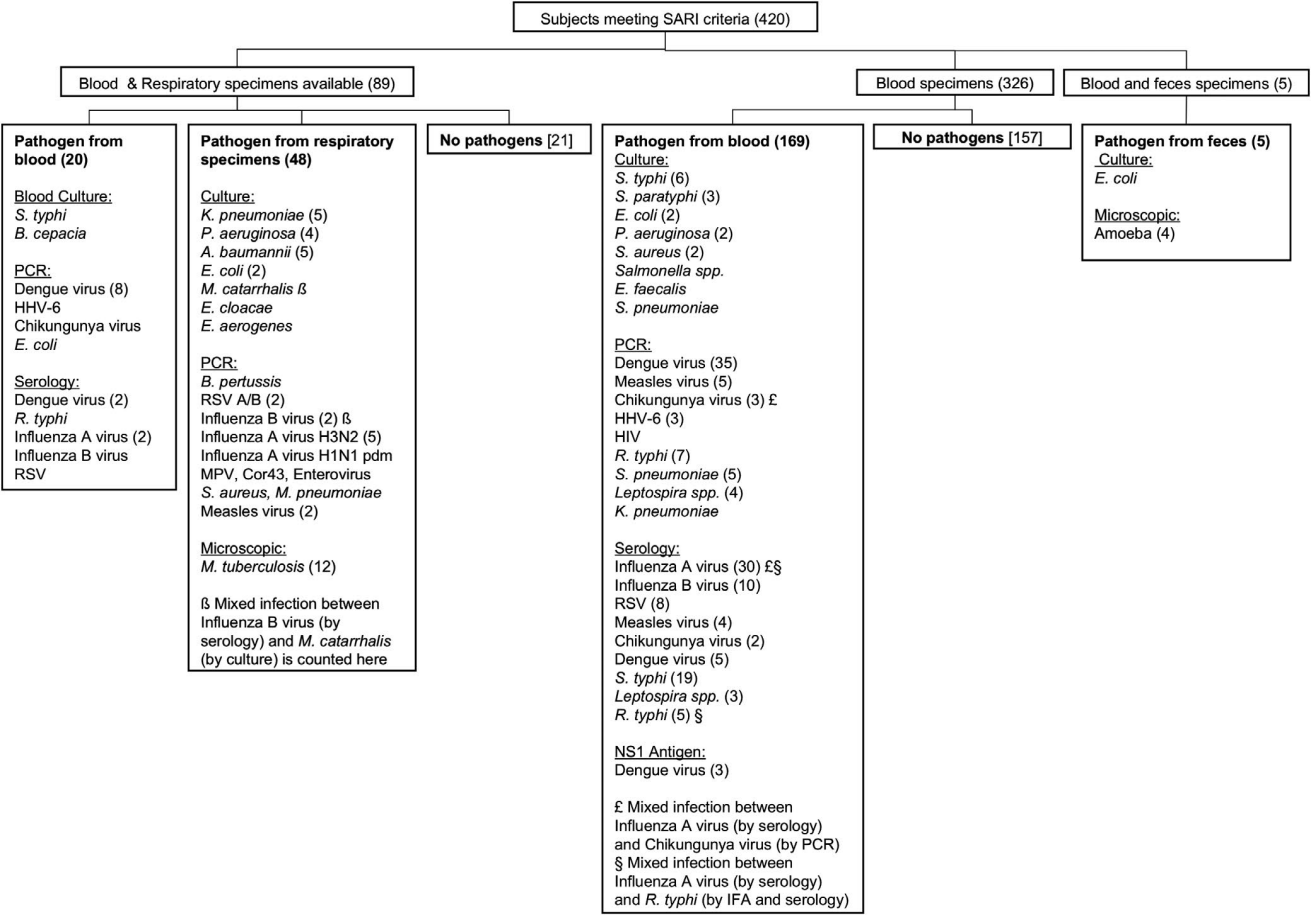
Study flow, specimen tested, and pathogen by diagnostic method. Urine and CSF are not included because no pathogens were identified from urine samples (n = 23) and CSF specimens were unavailable. The number of cases is shown in parenthesis when more than 1 case was identified

Diagnostic microbiology tests were conducted at hospitals immediately after specimens were collected and at the INA‐RESPOND laboratory retrospectively. The diagnostic laboratory evaluations and identified pathogens are shown in Figure 1. Details of the laboratory assays listed below and interpretation for each pathogen are listed in Table S1.

### 2.3 Hospital laboratory

At each hospital, all blood specimens, and other biological specimens, when available were cultured. Bacterial culture and identification were performed via VITEK® 2 (bioMérieux) and BD Phoenix™ (Becton‐Dickinson) automated systems, according to manufacturer instructions. Based on the standard of care, the two most common “systemic” pathogens, dengue virus and *Salmonella typhi,* were tested using rapid NS1 antigen and IgM/IgG antibody test; IgM *Salmonella typhi* was evaluated by Tubex TF. In a subset of cases, microscopic examination was performed on sputum or feces.

### 2.4 INA‐RESPOND laboratory testing

#### 2.4.1 “Systemic infection” pathogens

We used the term “systemic infections” to cover infections by several pathogens that are usually circulating in the blood and not commonly found in respiratory specimens, including dengue virus, *Salmonella spp., Rickettsia typhi, Leptospira spp.,* and chikungunya virus. Given that dengue is endemic in Indonesia, all blood specimens, regardless of the clinical presentation, were tested for dengue virus by real‐time polymerase chain reaction (rRT‐PCR), NS1 antigen ELISA, dengue IgM, and IgG ELISA assays. As other “systemic infections” are prevalent in Indonesia, they were tested when dengue testing was negative, using molecular and serological assays.

#### 2.4.2 Real‐time polymerase chain reaction for respiratory pathogens

Bacterial DNA was extracted from sputum or viral transport media (VTM) containing a respiratory swab using the QIAamp Bacterial DNA Mini Kit (Qiagen, Hilden) according to the manufacturer's protocol. Bacterial DNA was eluted in 100 µL of AE buffer and used as template for real‐time PCR assay or stored at −80°C. RNA was extracted from VTM containing a respiratory swab or sputum using the QIAamp Viral RNA Mini Kit (Qiagen, Hilden) according to the manufacturer's protocol. Viral RNA was eluted in 60 µL of AVE buffer and stored at −80°C.

#### 2.4.3 Detection of pathogens using real‐time PCR

Pathogen detection was performed with the QuantiTect Probe RT‐PCR Kit (QIAGEN; Cat#: 204443) in an Applied Biosystems 7500 Fast Real‐time PCR System (Thermo Fisher Scientific). The positive control was a synthetic plasmid carrying the nucleotide sequence of the detection target. Primers were synthesized and used to amplify the corresponding genome segments of influenza A and B, respiratory syncytial virus A and B, adenovirus, human metapneumovirus, parainfluenza virus 1, 2, 3, and 4, parechovirus, enterovirus, bocavirus, coronavirus, *Legionella pneumophila*, *Mycoplasma pneumoniae*, *Chlamydia pneumoniae*, *Chlamydophila psittaci*, *Haemophilus influenzae*, *Bordetella pertussis*, and *Streptococcus pneumoniae*.

### 2.5 Serology/ ELISA

Serologic testing was performed for influenza A, influenza B, respiratory syncytial virus (RSV), measles, and rubella. Influenza A (Cat# ESR1231M) and B (Cat# ESR1232M), virus IgM kits and influenza A (Cat# ESR1231G) and B (Cat# ESR1232G), and virus IgG kits (SERION ELISA classic kit Institut Virion/Serion GMBH‐Germany) were used according to manufacturer instructions. ELISA kits (Serion) were used to test IgM and IgG antibodies of RSV, measles, and rubella viruses. Since we used a semi‐quantitative ELISA method, we considered influenza/RSV/measles and rubella infection present when sero‐conversion or at least twofold increase of IgM and/or IgG titers was observed, consistent with manufacturer specifications and “Quality Standards in Microbiological/Infectiological Diagnostics.”^21^

### 2.6 Data analysis

Patient characteristics, clinical, hematology and chemistry data, and outcomes were collected via paper case report form, entered into the Open Clinica database, and tabulated according to SARI etiology. Pathogens were evaluated according to clinical diagnoses of respiratory and non‐respiratory conditions at discharge. Group comparisons were performed using chi‐square. Comparisons between continuous variables were performed by *t* test using STATA 17. Logistic regression was used to explore univariate relationships between the variables and influenza status. Regarding a multivariate model, we were faced with the issue of having a limited number of influenza cases relative to the number of variables under consideration. Thus, following guidance from Harrell (2015, Sections 4.3 and 4.7)^22^ for scenarios when there are a limited number of events (influenza cases) relative to the number of variables, we fit a multivariate logistic regression with LASSO penalty as an exploratory variable selection exercise. Statistical analyses were performed using SPSS version 22 (IBM Corporation) and R version 3.6.0.; the level of significance was set at *P* < .05. SARI criteria for diagnosing influenza were evaluated using randomly selected SARI and non‐SARI cases. Influenza PCR and/or serology assays were considered the gold standard.

## 3 RESULTS

Of 1464 subjects enrolled in the parent AFIRE study, 420 met the SARI criteria. Eighty‐nine had blood and respiratory specimens, and 331 had only blood specimens. The first screening for dengue and 4 other “systemic infections” (*Salmonella spp*., *Rickettsia spp*., chikungunya virus, and *Leptospira spp*.) contributed to 26% (109/420) of the total SARI cases. Influenza diagnostic tests were performed in all 420 SARI cases. Influenza was identified in 12.1% (51/420) cases, consisting of influenza A untypeable (32), influenza B (13), influenza A H3N2 (5), and influenza A H1N1 pdm (1). A total 43 out of 51 (83.7%) influenza cases were confirmed by serology only, including two cases that also had evidence of chikungunya virus and *Rickettsia typhi* infections, and 8 cases by RT‐PCR and serology including one mixed infection with *Moraxella catarrhalis*. Among 200 randomly selected SARI cases, 24 influenza cases were identified and among 200 randomly selected non‐SARI subjects, influenza was confirmed in 10 subjects, suggesting the sensitivity of SARI criteria to identify influenza cases was 70.6% (24/34), but the specificity was only 50.5% (190/366).

Other viral and bacterial respiratory pathogens contributed to 16.7% (70/420) of SARI cases, whereas non‐respiratory pathogens other than the five pathogens denoted above contributed to 3% (13/420) of cases. The most common respiratory viruses after influenza virus were RSV and measles (2.6% (11/420) each), whereas the most common bacteria were *Mycobacterium tuberculosis* (2.9% (12/420)), *Streptococcus pneumoniae, Pseudomonas aeruginosa,* and *Klebsiella pneumoniae*(1.4% (6/420) each). Pathogens and the specimen type used for diagnostic tests are listed in Figure 1. A total 178 out of 420 (42.4%) cases had no pathogen identified. In addition, identified pathogens in SARI and non‐SARI groups are listed in Table S2.

### 3.1 Identified pathogens based on clinical diagnoses

**Figure 2:**
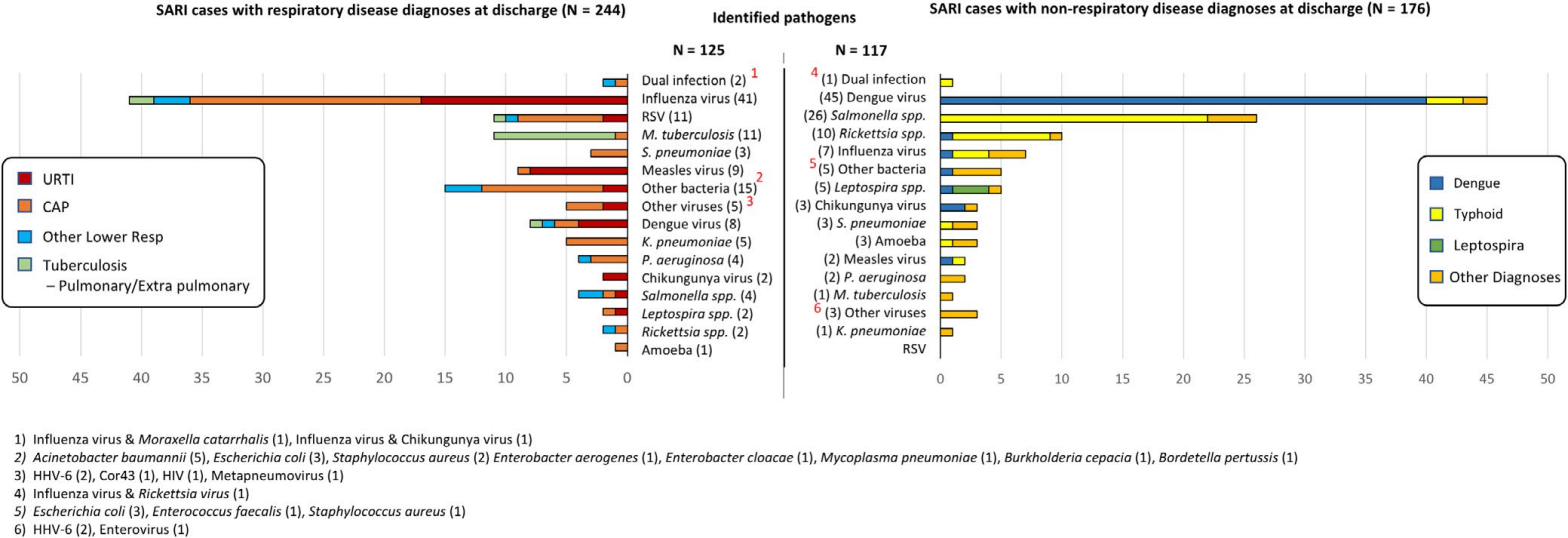
Identified pathogens based in clinical diagnoses

Among the 420 subjects who met SARI criteria, 244 (58.1%) had a hospital discharge diagnosis of respiratory disease and 176 (41.9%) of non‐respiratory disease. No subject had a discharge diagnosis of influenza. The three most common discharge diagnoses among patients considered to have respiratory diseases were pneumonia (124 (50.8%)), upper respiratory tract infection (68 (28%)), and pulmonary TB (24 (9.8%)). Dengue and typhoid fever were equally the most frequent discharge diagnosis among SARI patients with non‐respiratory diseases (55 (31.3%)) (Figure 2).

In this study, only 125 of the 244 subjects with a hospital discharge diagnosis of respiratory disease (51.2%) had identified causes, including influenza virus (41), other viruses (35), bacteria (46), mixed of influenza and bacteria (2), and parasite (1). Among 124 subjects diagnosed as having pneumonia, 59 (48%) had a confirmed etiology, consisting of 19 influenza cases, 13 other viral infections, 25 bacterial infections, 1 parasite, and 1 mixed influenza virus and bacteria; 65 remained unknown. In 68 clinically diagnosed URTI, influenza virus (17) and other viruses (10) were identified more frequently than bacteria (4) (Figure 2). Only 10/24 (41.7%) of the subjects with pulmonary TB as their discharge diagnosis were confirmed to have active *M. tuberculosis* infection with no other pathogens identified, while the rest of the causes were influenza (2) *Salmonella typhi* (2), dengue virus (1), RSV (1), and unidentified (8).

### 3.2 Demography, clinical manifestations, and hematological profiles

Unlike SARI cases associated with influenza that were distributed in all age groups, SARI associated with respiratory viruses was most common in children ≤5 years old, dengue in 5‐18 years old, and *S. typhi* and *R. typhi* in 5‐45 years old. The demography characteristics of SARI grouped by pathogens are listed in Table 1. Subjects with *S. typhi* and *R. typhi* were admitted to the hospitals significantly later compared to other groups. Among signs, symptoms, and hematological results, coryza was most common in respiratory viruses; headache, sore throat, vomiting, myalgia, arthralgia, leukopenia, thrombocytopenia, and lymphocytosis in dengue; chills, lethargy, abdominal pain, and diarrhea in rickettsiosis and typhoid fever; shortness of breath and leukocytosis in respiratory bacteria; and granulocytosis in non‐respiratory bacteria. Details of the proportion of these parameters in each pathogen group are listed in Table 1. and logistic regression in Table S3.

**Table 1:** Demographic, clinical, and laboratory characteristics by illness category amongst Indonesian SARI patients (N = 420)

Parameter		Dengue [N = 53]		Influenza [N = 48]		Salmonella and Rickettsia [N = 42]		Respiratory Bacteria^a^ [N = 41]		Respiratory Viruses^b^ [N = 29]		Other Bacteria^c^, [N = 16]		Unknown [N = 178]		*P‐*value^d^
Age	1‐<5 y.o., N (%)	8 (15.1)		11 (22.9)		4 (9.5)		3 (7.3)		**20 (69.0)**		1 (6.3)		54 (30.3)		<.001
	5‐<18 y.o., N (%)	**26 (49.1)**		9 (18.8)		16 (38.1)		4 (9.8)		3 (10.3)		3 (18.8)		38 (21.3)		<.001
	18‐<45 y.o., N (%)	16 (30.2)		14 (29.2)		16 (38.1)		15 (36.6)		5 (17.2)		3 (18.8)		50 (28.1)		.462
	45‐<60 y.o., N (%)	1 (1.9)		8 (16.7)		4 (9.5)		8 (19.5)		1 (3.4)		2 (12.5)		17 (9.6)		.066
	≥60 y.o., N (%)	2 (3.8)		6 (12.5)		2 (4.8)		11 (26.8)		0 (0)		**7 (43.8)**		19 (10.7)		<.001
Male gender, N (%)		27 (50.9)		25 (52.1)		20 (47.6)		27 (65.9)		18 (62.1)		7 (43.8)		90 (50.6)		.517
Clinical symptoms	Shortness of breath, N (%)		**4 (7.5)**		21 (43.8)		9 (21.4)		**33 (80.5)**		15 (51.7)		8 (50.7)		84 (47.2)	<.001
	Haemoptysis, N (%)		0 (0)		3 (6.3)		0 (0)		1 (2.4)		0 (0)		0 (0)		5 (2.8)	.313
	Coryza, N (%)		19 (35.8)		18 (37.5)		11 (26.2)		**3 (7.3)**		**20 (69.0)**		2 (12.5)		67 (37.6)	<.001
	Sore throat, N (%)		**21 (39.6)**		9 (18.8)		6 (14.3)		3 (7.3)		2 (6.9)		3 (18.8)		25 (36.2)	<.001
	Chills, N (%)		4 (7.5)		12 (25.0)		**15 (35.7)**		5 (12.2)		2 (6.9)		5 (31.3)		24 (13.5)	.001
	Lethargy, N (%)		18 (34.0)		12 (25.0)		**22 (52.4)**		16 (39.0)		5 (17.2)		8 (50.0)		48 (27.0)	.007
	Vomiting, N (%)		**19 (35.8)**		8 (16.7)		9 (21.4)		1 (2.4)		1 (3.4)		4 (25.0)		11 (6.2)	<.001
	Diarrheal, N (%)		4 (7.5)		1 (2.1)		**7 (16.7)**		0 (0)		0 (0)		2 (12.5)		4 (2.2)	<.001
	Myalgia, N (%)		**10 (18.9)**		3 (6.3)		2 (4.8)		1 (2.4)		0 (0)		3 (18.8)		8 (4.5)	.002
	Arthralgia, N (%)		**14 (26.4)**		3 (6.3)		6 (14.3)		0 (0)		0 (0)		2 (12.5)		9 (5.1)	<.001
	Abdominal pain, N (%)		12 (22.6)		4 (8.3)		**15 (35.7)**		3 (7.3)		1 (3.4)		3 (18.8)		24 (13.5)	.001
	Headache, N (%)		**29 (54.7)**		13 (27.1)		17 (40.5)		3 (7.3)		2 (6.9)		6 (37.5)		29 (16.3)	<.001
Fatal cases, N (%)		1 (1.9)		2 (4.2)		3 (7.1)		8 (19.5)		1 (3.4)		1 (6.3)		17 (9.6)		.054
Day of onset, (Mean ± SD)		4.1 ± 1.5		4.7 ± 3.1		**6.8 ± 3.5**		5.5 ± 4.0		4.8 ± 2.7		4.6 ± 2.4		4.5 ± 2.8		<.001
Haematology profile	Haemoglobin (g/dL), (Mean ± SD)	12.92 ± 1.75		**13.10 ± 2.00**		12.69 ± 1.84		**11.49 ± 2.40**		11.57 ± 1.95		12.06 ± 1.61		11.92 ± 2.24		<.001
	Haematocrit (%), (Mean ± SD)	38.08 ± 5.47		38.37 ± 5.45		37.17 ± 5.12		**34.41 ± 6.12**		35.61 ± 5.06		35.75 ± 4.57		35.62 ± 6.43		.005
	Leukocyte(×10^3^/mm^3^), (Mean ± SD)	**5.50 ± 3.99**		12.19 ± 6.65		7.62 ± 4.07		**15.20 ± 9.19**		12.32 ± 7.98		12.31 ± 6.59		12.68 ± 7.18		<.001
	Platelet (×10^3^/mm^3^), (Mean ± SD)	**139.89 ± 97.09**		243.35 ± 107.30		171.62 ± 77.82		**306.54 ± 169.14**		294.61 ± 124.14		197.00 ± 138.44		262.18 ± 126.31		<.001
	Granulocyte (%), (Mean ± SD)	**59.31 ± 18.49**		74.36 ± 14.80		66.18 ± 13.44		76.64 ± 17.32		63.00 ± 13.85		**83.08 ± 8.12**		71.96 ± 15.39		<.001
	Lymphocyte (%), (Mean ± SD)	**29.93 ± 18.23**		16.98 ± 13.10		25.39 ± 11.95		12.79 ± 9.88		28.27 ± 12.96		**11.75 ± 8.31**		19.86 ± 13.33		<.001

Note

*Heat Map Index:* 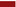 >50%, 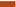 40%‐50%, 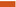 30%‐40%, 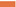 20%‐30%, 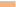 10%‐20%, 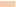 1%‐10%.

Heat map of patient characteristics by etiology of illness.

^a^ Respiratory Bacteria; *S pneumoniae* (6), *M tuberculosis* (12), *K pneumoniae* (6), *M pneumoniae* (1), *A baumannii* (5), *P cepacia* (1), *P aeruginosa* (6), *S aureus* (3). *B pertussis* (1).

^b^ Respiratory Viruses; Cor‐43 (1), Enterovirus (1), HHV‐6 (4), Measles virus (11), hMPV (1), RSV (11).

^c^ Other Bacteria; *Leptospira*spp. (7), *E coli* (6), *E aerogenes*, *E cloacae*, *E* *faecalis* (@1).

^d^ The chi‐square or ANOVA test were used to assess differences in frequency/mean amongst etiologies. Cells in bold show the highest or lowest percentage/value of parameters in each category when the mean difference was significant at the .05 level compared with two or more other categories by pairwise comparisons post‐hoc tests. Other Viruses (Chikungunya virus (5), HIV (1)), Amoeba (4), and co‐infection cases (3) were not included in the analysis since N < 10 in those each group.

### 3.3 Comorbidities and outcomes

The proportion of SARI in hospitalized patients with fever was higher in ≤5 years old and ≥60 years old (52.7% and 48.6%) than in the 5 to ≤18 years old and 18 to <60 years old groups (25.5% and 22.3%, respectively) (Table 2). However, the mortality rate among SARI cases was highest in ≥60 years and 45 to <60 years old groups (22.4% and 21.4%, respectively). Fatalities occurred more frequently in bacterial respiratory pathogens (19.5%) compared to influenza (6.3%) groups. Among these fatal cases, 27 were diagnosed as respiratory diseases and 8 were diagnosed as non‐respiratory diseases at discharge. Most fatal cases were associated with mono‐ or co‐infection of *Mycobacterium tuberculosis* and HIV. Only 3 of the 35 (8.6%) fatal cases did not have any underlying diseases.

**Table 2:** Pathogen identified and outcome based on age group

	Age group					
	1‐<5 y.o.	5‐<18 y.o.	18‐<45 y.o.	45‐<60 y.o.	≥60 y.o.	Total
Enrolled subjects	198	409	585	167	105	1464
Subjects with SARI	104	106	119	42	49	420
Fatal cases, N(%)	4 (3.8%)	3 (2.8%)	8 (6.7%)	9 (21.4%)	11 (22.4%)	35 (8.3%)
Pathogens identified:						
Non‐respiratory pathogens, N (^a^)						
Dengue virus	8	26	16	1	2 (1)	53 (1)
*Salmonella*spp.	3	14	11 (1)	2 (1)	0	30 (2)
*Rickettsia typhi*	1	2	5	2	2 (1)	12 (1)
*Leptospira*spp.	0	2	2	2	1	7
Chikungunya virus	1	4	0	0	0	5
*Escherichia coli*	0	1	0	0	5 (1)	6 (1)
Amoeba	2	1	0	0	1	4
*Enterobacter aerogenes*	0	0	1	0	0	1
*Enterobacter cloacae*	0	0	0	0	1	1
*Enterococcus faecalis*	1	0	0	0	0	1
HIV	0	1 (1)	0	0	0	1 (1)
Respiratory pathogens, N (^a^)						
Influenza virus^b^	11	10^b^	14	9^b^ (3)	7^b^	51 (3)
RSV	9	1	0	1 (1)	0	11 (1)
Measles virus	4	2	5	0	0	11
*Mycobacterium tuberculosis*	0	0	6 (3)	3	3 (2)	12 (5)
*Klebsiella pneumoniae*	1	0	2	1	2	6
*Streptococcus pneumoniae*	1 (1)	1	1	2	1	6 (1)
HHV‐6	4	0	0	0	0	4
*Bordetella pertussis*	0	1	0	0	0	1
Cor43	1	0	0	0	0	1
Metapneumovirus	1	0	0	0	0	1
*Mycoplasma pneumoniae*	0	0	0	1	0	1
Enterovirus	1	0	0	0	0	1
*Pseudomonas aeruginosa*	1	0	3 (1)	1	1	6 (1)
*Staphylococcus aureus*	0	1	2	0	0	3
*Acinetobacter baumannii*	0	1	1	0	3 (1)	5 (1)
*Burkholderia cepacia*	0	0	0	0	1	1
Unknown	54 (3)	38 (2)	50 (3)	17 (4)	19 (5)	178 (17)

^a^ Number of fatal case.

^b^ Three cases mixed infection with *Moraxella catarrhalis, Rickettsia typhi,* and chikungunya virus.

Three influenza cases were also fatal. The first was a 49‐year‐old woman with rheumatic heart disease, the second was a 49‐year‐old woman with bronchiectasis, and the third was 59‐year‐old woman with mediastinal tumor and heart disease.

Among the SARI subjects, 173 (41.2%) had one or more comorbidities. These were more common in subjects diagnosed with respiratory diseases (132/244 (54.1%)) compared to subjects diagnosed as having non‐respiratory diseases (39/176 (22.1%)).

## 4 DISCUSSION

The prevalence of influenza within SARI patients in our cohort (12.1%) was consistent with previous findings from Indonesia. A 2011 study across nine hospitals reported a prevalence of 6%^23^ and a 2013‐2016 study across three hospitals reported a prevalence of 14%, suggesting the annual incidence of influenza‐associated SARI was 13‐19 per 100,000 population.^18^ Similar proportions of influenza among SARI cases have been reported in other parts of the world, 11.8% in Middle Eastern countries in 2007‐2014,^24^ and 12% in China in 2010‐2012.^25^ Our study confirms that influenza is an important cause of hospitalization in Indonesia. Since influenza was never diagnosed during hospitalization, our findings highlight the need for improved diagnostic strategies, optimization of management, and a national influenza vaccination program in Indonesia, now only mandatory for pilgrims.

Sensitivity and specificity of SARI criteria to identify influenza in our study are comparable with other studies from Western Kenya and Canada (72.1% vs 65.3%‐84% for sensitivity and 29.5% vs. 22.5%‐24.7% for specificity). In contrast, a study from Northern India reported a sensitivity and specificity of 37% and 78%, respectively.^14^ These disparate results may be due to use of the previous SARI criteria by the latter study. The low specificity of SARI criteria seems related to its broad clinical criteria, resulting in 176 (41.9%) cases of non‐respiratory illness meeting the definition of SARI.

“Systemic” viral and bacterial infections may manifest clinically with respiratory signs and/or may progress to complicated pneumonia. Dengue with respiratory manifestations has been reported in more than 60% of the schoolchildren in Colombia,^26^ in 4 Taiwanese patients, and 1 returned traveler in the United States, whose nasopharyngeal swabs were also positive by RT‐PCR.^5^, ^6^ Dengue was also the third leading cause of acute respiratory distress syndrome in pediatric patients.^27^ Similarly, leptospirosis may present with severe pulmonary manifestations^10^, ^28^ and the bacteria can be detected from throat swabs.^10^ Other “systemic” diseases such as chikungunya, rickettsiosis, and typhoid fever may also present with respiratory manifestations.^8^, ^9^, ^11^ Although SARI patients with discharge diagnoses of such “systemic” diseases constituted a large proportion of cases, missed diagnoses only occurred in 25% (27/109) because in the majority of cases, clinicians were able to make a diagnosis based on other clinical manifestations (eg, diarrhea, rash, and icterus) and hematology profiles (eg, leukopenia, thrombocytopenia, and granulocytosis).

Physicians should be aware that many diseases can mimic Influenza and engender diagnostic confusion as demonstrated in our study. In our study, none of clinical and hematological profiles can be used to distinguish influenza and infections by other pathogens.

Identification of dengue as the most frequent cause of SARI may simply reflect the high rates of dengue in the AFIRE cohort. The 53 dengue cases that met SARI criteria were only 11.3% of the total dengue cases in the AFIRE study. Identification of Influenza as the most common virus may also be biased as serological tests were only available and performed for influenza, RSV, and measles. However, the misdiagnosis of influenza in our cohort and the discharge diagnosis of a non‐respiratory illness in 7/51 (13.7%) confirmed influenza cases are concerning. Clinicians in Indonesia may need education about influenza infection to mitigate misdiagnoses.^29^ Public health campaigns and continuing medical education will be important in this regard. Healthcare policymakers should consider implementing SARI criteria to screen for Influenza in Indonesia given the sensitivity of 70.6%.

Only 57.6% (242/420) of cases had an identified etiology, consistent with other studies and suggesting the need for improved diagnostic approaches. A study of ILI in the ICU setting identified at least one pathogen in 45% of ILI subjects, with 75.2% of those cases having only a single pathogen.^30^ Laboratory capacity, specimen collection technique and handling, communication among multidisciplinary team members, and recognition of the appropriate differential diagnosis are critical for facilitating pathogen identification. Rapid influenza testing has low sensitivity (50%‐70%), while molecular diagnosis requires trained technicians and specialized instruments.^31^ We herein confirmed that serologic testing as an adjunct to RT‐PCR was helpful for identification of viral pathogens in community‐acquired pneumonia.^32^

By improving clinician awareness of influenza and SARI, diagnostic capacity, influenza vaccination coverage and targeted public health policy, etiology of illness among patients presenting to hospital with SARI is more likely to be elucidated. This will facilitate appropriate treatment, which could lead to shorter hospital stays and decrease treatment costs.^33^, ^34^ Identification of SARI etiology could also reduce inappropriate antibiotic use, thus minimizing the emergence of the antimicrobial resistance.^35^, ^36^, ^37^, ^38^ Etiologic diagnosis is also important for controlling transmission and preventing outbreaks.^39^, ^40^ For example, prevention strategies such as respiratory isolation or cohorting when individual rooms are unavailable could be implemented. One study reported that the RR of hospital‐acquired ILI was 5.4 for patients who had contact with at least one infectious health worker, 17.96 for patients with contact with at least one infectious patient, and 34.75 for those with contact with at least one infectious patient and one infectious health worker.^41^

This study had several limitations. It was not designed specifically for identification of influenza or SARI. Thus, respiratory specimens were only collected from a subset of patients with diagnosis of respiratory diseases, suggesting that we may have missed some influenza cases although influenza serological test was performed in acute and convalescent plasma of all of these subjects. For serology, we also used a twofold increase in ELISA IgM/IgG titers as the criteria for acute influenza infection instead of the fourfold increase in traditional titer^21^ assays that would increase the sensitivity at the expense of some false positives. However, it has been reported that increase in antibodies’ titers after infection ranges from 1.2‐ to 10.2‐fold and 39%–55% of infected persons would not have a fourfold or greater rise in antibody titer after infection.^42^ As such, this group may be different from the group that had respiratory specimens, limiting generalizability of findings.

The high proportion of cases without an identified etiology also suggests that pathogen distribution might be different if etiologies were identified in every case. Although unavailability of specimens may have limited ability to identify an etiology, our rates of pathogen identification are consistent with those of other studies. In addition, this was an observational study conducted at 8 tertiary hospitals with varied clinical practices and patient populations, which can introduce bias.

A major strength of this study was testing for a complete panel of respiratory viral and bacterial pathogens by culture, molecular assays, and serological tests on acute and convalescent plasma. In addition, this study is the first to report the epidemiology of non‐respiratory pathogens associated with SARI and the first to evaluate SARI criteria for identifying influenza cases in Indonesia.

In conclusion, influenza is often overlooked as an etiology of febrile illness in Indonesia. Implementation of the SARI criteria in tertiary referral hospitals would help identify potential influenza infections. Public health strategies to address the high burden of influenza should include vaccination programs, clinician education and awareness campaigns, improved diagnostic capacity, and support for effective point‐of‐care tests.

## CONFLICT OF INTEREST

The authors have declared that no competing interests exist.

## AUTHOR CONTRIBUTIONS

**Abu Tholib Aman:** Conceptualization (equal); Data curation (equal); Formal analysis (equal); Investigation (equal); Methodology (equal); Project administration (equal); Supervision (equal); Writing‐original draft (equal); Writing‐review & editing (equal). **Tri Wibawa:** Data curation (equal); Investigation (equal); Methodology (equal); Resources (equal); Supervision (equal); Writing‐review & editing (equal). **Herman Kosasih:** Conceptualization (equal); Formal analysis (equal); Methodology (equal); Supervision (equal); Validation (equal); Writing‐original draft (lead); Writing‐review & editing (equal). **Rizka Humardewayanti Asdie:** Data curation (equal); Investigation (equal); Resources (equal). **Ida Safitri:** Data curation (equal); Investigation (equal); Resources (equal); Writing‐review & editing (equal). **Umi Solekhah Intansari:** Data curation (equal); Investigation (equal); Resources (equal); Writing‐review & editing (equal). **Yuli Mawarti:** Data curation (equal); Investigation (equal); Resources (equal); Writing‐review & editing (equal). **Pratiwi Sudarmono:** Conceptualization (equal); Data curation (equal); Investigation (equal); Methodology (equal); Project administration (equal); Resources (equal); Writing‐review & editing (equal). **Mansyur Arif:** Conceptualization (equal); Data curation (equal); Investigation (equal); Methodology (equal); Project administration (equal); Resources (equal); Writing‐review & editing (equal). **Dwiyanti Puspitasari:** Data curation (equal); Investigation (equal); Resources (equal); Writing‐review & editing (equal). **Bachti Alisjahbana:** Conceptualization (equal); Data curation (equal); Investigation (equal); Methodology (equal); Project administration (equal); Supervision (equal); Writing‐review & editing (equal). **Ketut Tuti Merati Parwati:** Conceptualization (equal); Data curation (equal); Investigation (equal); Methodology (equal); Project administration (equal); Supervision (equal); Writing‐review & editing (equal). **Muhammad Hussein Gasem:** Conceptualization (equal); Data curation (equal); Investigation (equal); Methodology (equal); Project administration (equal); Resources (equal); Supervision (equal); Writing‐review & editing (equal). **Dewi Lokida:** Conceptualization (equal); Data curation (equal); Investigation (equal); Methodology (equal); Resources (equal); Writing‐review & editing (equal). **Nurhayati Lukman:** Data curation (equal); Investigation (equal); Supervision (equal); Writing‐review & editing (equal). **Teguh Sarry Hartono:** Data curation (equal); Investigation (equal); Supervision (equal); Writing‐review & editing (equal). **Yan Mardian:** Data curation (equal); Formal analysis (equal); Visualization (equal); Writing‐review & editing (equal). **C Jason Liang:** Formal analysis (equal); Methodology (equal); Software (equal); Validation (equal); Visualization (equal); Writing‐original draft (equal); Writing‐review & editing (equal). **Sophia Siddiqui:** Conceptualization (equal); Methodology (equal); Writing‐review & editing (equal). **Muhammad Karyana:** Conceptualization (equal); Investigation (equal); Methodology (equal); Project administration (equal); Resources (equal); Supervision (equal); Writing‐original draft (equal). **Chuen‐Yen Lau:** Supervision (equal); Validation (equal); Visualization (equal); Writing‐review & editing (equal).

## AUTHORS’ CONTRIBUTION

ATA, HK, PS, MA, BA, TP, MHG, DL, MK, and SS designed the study and prepared the protocol. MK, MHG, TW, RH, IS, USI, YM, DP, BA, ATA, DL, PS, TP, MA, HK, N, and TSH conducted and supervised the study. MK, DL, CJL, HK, YM, TW, RH, YM, ATA, SS, and CYL analyzed the data and prepared the draft of the manuscript. All reviewed and finalized the manuscript.

## Supporting information

Tables S1‐S3
