# Supplementary material for: Etiologies of severe acute respiratory infection (SARI) and misdiagnosis of influenza in Indonesia, 2013‐2016
Source: Influenza Other Respir Viruses. 2020 Jul 14;15(1):34–44. doi: 10.1111/irv.12781 (PMC7405185; doi:10.1111/irv.12781)
Supplement: Supplementary file 1 — Tables S1‐S3 [file IRV-15-34-s001.docx]

Supplement Table 1. Molecular and serology tests at the INA-RESPOND Laboratory

| **Pathogen** | **Methods** | **Confirmation** |
| --- | --- | --- |
| Dengue virus | Molecular: real-time PCR^1^ and nested PCR^2^ | DENV RNA was detected in plasma |
|  | NS1 Antigen detection (Focus Technology, US) | NS1 antigen was detected in plasma |
|  | Serology assay: ELISA IgM and IgG (Focus Technology, US) | Sero-conversion or increase index in IgM and or IgG between acute and convalescent specimens |
| *Salmonella typhi/ Salmonella paratyphi* | Blood culture (Bactec or ViTek) | Identified by Vitek/Phoenix |
|  | Molecular: *S. typhi*^3^, *S. paratyphi*^4^ | *S. typhi/S. paratyphi* DNA was detected in buffy coat |
|  | *S. typhi* antibodies by ELISA (MyBioSource, US) IgM and IgG | Sero-conversion or increasing of IgM and/or IgG antibodies titer. |
| *Rickettsia typhi* | Molecular: *Rickettsia spp.*^5^, *R. typhi*^6^ | *R. typhi* DNA was detected in plasma or buffy coat |
|  | Murine typhus IFA (Focus, US) | Increase in Fluorescein level in convalescent specimens, consistent with ELISA results |
| Leptospira | Micro agglutination test (MAT) in a few specimens | Four-fold increase of Leptospira antibodies to certain species, or a high titer (1/320) of antibodies in a single acute specimen |
|  | Molecular: real time PCR^7^ | Leptospira DNA was detected in plasma |
|  | ELISA IgM leptospira (PanBio, Aus) | Sero-conversion or increasing IgM index |
|  |  |  |
| Chikungunya virus | Molecular: real time PCR^8^ | Chikungunya RNA was detected in plasma |
|  | ELISA IgM and IgG chikungunya antibodies (Euroimmune, Germany) | Sero-conversion or increasing titers of IgM and IgG in convalescent specimens |
|  |  |  |
| Influenza virus | Molecular: multiplex real-time PCR^9^ | Influenza RNA virus was detected in swab or sputum |
|  | ELISA Influenza A and B virus IgM and IgG antibodies (Serion, Germany) | At least two fold increase of IgM and/or IgG influenza A or Influenza B antibodies in convalescent specimens |
|  |  |  |
| Respiratory syncytial virus | Molecular: multiplex real-time PCR^9^ | RSV RNA was detected in respiratory specimens |
|  | ELISA RSV IgM and IgG antibodies (Serion, Germany) | Sero-conversion or increasing of IgM or IgG RSV antibodies OD in convalescent specimens |
| Cor OC43 | Molecular: multiplex real-time PCR^9^ | CorOC43 virus RNA was detected in respiratory specimens |
|  |  |  |
| Enterovirus | Real time PCR^13^ | Enterovirus RNA is detected |
| Adenovirus | Molecular: Multiplex real time PCR^9^ | Adenovirus RNA is detected |
| Measles virus | Molecular: conventional^10^  ELISA measles virus IgM and IgG antibodies (Serion, Germany) | Measles DNA was detected in sputum/swab or plasma  Sero-conversion or increase titers of IgM and/or IgG antibodies in convalescent specimens |
|  |  |  |
| HIV | Real-time PCR^18^  Rapid Test SD^®^, Oncoprobe^®^  ELISA 4^th^ generation (Biorad^®^) | HIV RNA was detected, HIV antibody was negative,  4^th^ generation ELISA was positive |
|  |  |  |
| Metapneumovirus | Molecular: multiplex real-time PCR^9^ | Metapneumovirus RNA was detected in swab/sputum |
|  |  |  |
| HHV-6 | Conventional PCR^11^ | HHV-6 virus DNA was detected in plasma |
|  | Real-time PCR^12^ | >1,000 copies/ul |
| *Mycobacterium tuberculosis* | Acid fast bacilli smear (microscopic) | *M. tuberculosis* bacilli was detected in sputum |
|  |  |  |
| *Amoeba coli/*  *Amoeba histolytica* | Microscopic examination | Amoeba was detected in faeces |
|  |  |  |
| *Escherichia coli*  *Streptococcus pneumoniae* | Blood or sputum culture  Real time PCR  Blood culture  Real-time PCR^14^ | Identified by Vitek/Phoenix  *E. coli* DNA was identified in plasma  Identified by Vitek/Phoenix  *S. pneumoniae* DNA was detected in blood |
|  |  |  |
| *Bordetella pertussis* | Real-time PCR^15^ | *B. pertussis* DNA was detected in respiratory specimens |
|  |  |  |
| *Mycoplasma pneumoniae* | Real-time PCR^16^ | *M. pneumoniae* DNA was detected in respiratory specimens |
| *Staphylococcus aureus* | Real-time PCR^17^  Blood culture | *S. aureus* DNA was detected in respiratory specimens  Identified by Vitek/Phoenix |
| *Klebsiella pneumoniae*  *Moraxella catarrhalis*  *Burkholderia cepacia*  *Pseudomonas aeruginosa*  *Acinetobacter baumannii*  *Enterobacter aerogenes*  *Enterococcus faecalis*  *Enterobacter cloacae* | Real-time PCR^17^  Sputum culture  Sputum culture  Blood culture  Sputum or blood culture  Sputum culture  Sputum culture  Blood culture  Sputum culture | *K. pneumoniae* DNA was detected in plasma  Identified by Vitek/Phoenix  Identified by Vitek/Phoenix  Identified by Vitek/Phoenix  Identified by Vitek/Phoenix  Identified by Vitek/Phoenix  Identified by Vitek/Phoenix  Identified by Vitek/Phoenix  Identified by Vitek/Phoenix |
|  |  |  |

Supplement 1: Testing performed for each pathogen and results considered indicative or a positive result are shown.

**Footnotes:**

1. Hue KD, Tuan TV, Thi HT, et al. Validation of an internally controlled one-step real-time multiplex RT-PCR assay for the detection and quantitation of dengue virus RNA in plasma. *J Virol Methods* 2011; **177**(2):168–73.

2. Lanciotti RS, Calisher CH, Gubler DJ, Chang GJ, Vorndam AV. Rapid detection and typing of dengue viruses from clinical samples by using reverse transcriptase-polymerase chain reaction. J Clin Microbiol 1992; 30(3):545–51.

3. Hatta M, Smits HL. Detection of Salmonella typhi by nested polymerase chain reaction in blood, urine, and stool samples. Am J Med Trop Med Hyg 2007; 76(1):139–43.

4. Pratap CB, Kumar G, Patel SK, et al. Mix-infection of S. typhi and paratyphi A in typhoid fever and chronic typhoid carriers: a nested PCR based study in North India. J Clin Diagn Res 2014; 8(11):DC09–DC14.

5. Jiang J, Chan TC, Temenak JJ, Dasch GA, Ching WM, Richards AL. Development of a quantitative real-time polymerase chain reaction assay specific for Orientia tsutsugamushi. Am J Med Trop Med Hyg 2004; 70(4):351–6.

6. Henry KM, Jiang J, Rozmajzl PJ, Azad AF, Macaluso KR, Richards AL. Development of quantitative real-time PCR assays to detect Rickettsia typhi and Rickettsia felis, the causative agents of murine typhus and flea-borne spotted fever. Mol Cell Probes 2007; 21(1):17–23.

7. Thaipadungpanit J, Chierakul W, Wuthiekanun V, et al. Diagnostic accuracy of real-time PCR assays targeting 16S rRNA and lipL32 genes for human leptospirosis in Thailand: a case-control study. PLoS One 2011; 6(1).

8. Lanciotti RS, Kosoy OL, Laven JJ, et al. Chikungunya virus in US travelers returning from India, 2006. Emerg Infect Dis 2007; 13(5):764–7.

9. Jansen RR, Schinkel J, Koekkoek S, et al. Development and evaluation of a four-tube real time multiplex PCR assay covering fourteen respiratory viruses, and comparison to its corresponding single target counterparts. Clin Diagn Virol 2011; 51(3):179–85.

10. Chibo D, Birch CJ, Rota PA, Catton MG. Molecular characterization of measles viruses isolated in Victoria, Australia, between 1973 and 1998. J Gen Virol 2000; 81(Pt 10):2511–8.

11. Huang LM, Kuo PF, Lee CY, Chen JY, Liu MY, Yang CS. Detection of human herpesvirus-6 DNA by polymerase chain reaction in serum or plasma. J Med Virol 1992; 38(1):7–10.

12. Sedlak RH, Cook L, Huang ML, et al. Identification of chromosomally integrated human herpesvirus 6 by droplet digital PCR. Clin Chem 2014; 60(5):765–72.

13. Beld M, Minnaar R, Weel J, et al. Highly sensitive assay for detection of enterovirus in clinical specimens by reverse transcription-PCR with an armored RNA internal control. J Clin Microbiol 2004; 42(7):3059–64.

14. Corless CE, Guiver M, Borrow R, Edwards-Jones V, Fox AJ, Kaczmarski EB. Simultaneous Detection of Neisseria meningitidis, Haemophilus influenzae, and Streptococcus pneumoniae in suspected cases of meningitis and septicemia using real-time PCR. J Clin Microbiol 2001; 39(4):1553–8.

15. Reischl U, Lehn N, Sanden GN, Loeffelholz MJ. Real-time PCR assay targeting IS481 of Bordetella pertussis and molecular basis for detecting Bordetella holmesii. J Clin Microbiol 2001; 39(5):1963–6.

16. Pitcher D, Chalker VJ, Sheppard C, George RC, Harrison TG. Real-time detection of Mycoplasma pneumoniae in respiratory samples with an internal processing control. J Med Microbiol 2006; 55:149–55.

17. Gadsby NJ, McHugh MP, Russell CD, Mark H, Conway Morris A, Laurenson IF, Hill AT,  Templeton KE.  2015. Development of two real-time multiplex PCR assays for the detection and quantification of eight key bacterial pathogens in lower respiratory tract infections. Clin Microbiol Infect 2015; 21: 788.e1–788.e13.

18. Palmer S, Wiegand AP, Maldarelli F, et al. New real-time reverse transcriptase-initiated PCR assay with single-copy sensitivity for Human Immunodeficiency Virus type 1 RNA in plasma. J Clin Microbiol 2003; 41(10):4531–6.

Supplement Table 2. Pathogen identified in SARI and non-SARI cases

|  | **SARI criteria (N=420)** | **NON-SARI criteria (N=200)** |
| --- | --- | --- |
| **Non-respiratory pathogens** |  |  |
| Dengue virus | 53 | 82* |
| *Salmonella* | 29 | 19 |
| *Rickettsia typhi* | 12 | 20 |
| *Leptospira* | 7 | 10 |
| Chikungunya virus | 6 | 7 |
| *Escherichia coli* | 6 | 3 |
| Amoeba | 4 | 1 |
| *Enterobacter aerogenes* | 1 | 0 |
| *Enterobacter cloacae* | 1 | 0 |
| *Enterococcus faecalis* | 1 | 2 |
| HIV | 1 | 0 |
| Hepatitis A virus | 0 | 1 |
| Seoul Virus | 0 | 1 |
| **Total** | **121** | **146** |
| **Respiratory pathogens** |  |  |
| Influenza virus | 51** | 10*** |
| RSV | 11 | 0 |
| Measles virus | 11 | 0 |
| *Mycobacterium tuberculosis* | 12 | 0 |
| *Klebsiella pneumoniae* | 6 | 1 |
| *Streptococcus pneumoniae* | 6 | 1 |
| HHV-6 | 4 | 1 |
| *Bordetella pertussis* | 1 | 0 |
| Cor43 | 1 | 0 |
| Metapneumovirus | 1 | 0 |
| *Mycoplasma pneumoniae* | 1 | 0 |
| Adenovirus | 0 | 0 |
| Enterovirus | 1 | 0 |
| *Pseudomonas aeruginosa* | 6 | 0 |
| *Staphylococcus aureus* | 3 | 0 |
| *Acinetobacter baumannii* | 5 | 0 |
| *Burkholderia cepacia* | 1 | 0 |
| **Total** | **121** | **13** |
| **Unknown** | 178 | 41 |

Supplement 2: Distribution of cases with identified pathogens is shown for patients who met SARI criteria and those who did not meet SARI criteria. *1 case mixed infection of dengue virus and *E faecalis* **3 cases of mixed infection of Influenza and *Moraxella catarrhalis, Rickettsia typhi,* or chikungunya.***7 cases of mixed infection of influenza and dengue virus (4), *R typhi* (1), *S typhi* (1), and *S pneumoniae* (1)

Supplement Table 3. Demographic, Clinical, and Laboratory Characteristics of SARI Patients with Influenza only [N=48] vs. others SARI patients [N=372]

| **Variables** | **SARI –Influenza (N=48)** | **SARI – non-Influenza (N=372)** | **Univariate Logistic Regression** | | | **Multivariate Logistic Regression (LASSO penalty)** | |
| --- | --- | --- | --- | --- | --- | --- | --- |
|  |  |  | **Odds Ratio** | **95% CI** | **p-value** | | **Odds Ratio** |
| Age, Median (IQRs) | 19 (7 – 46) | 17 (5 – 37) | 1.006 | 0.994 – 1.019 | 0.320 | |  |
| Gender, N (%)   - Male - Female | - 25 (51.1) - 23 (47.9) | - 173 (46.5) - 199 (53.5) | 0.945 | 0.518 – 1.725 | 0.854 | |  |
| Duration of Illness, Median (IQRs) | 4 (3 – 4) | 4 (3 – 6) | 0.985 | 0.888 – 1.093 | 0.778 | |  |
| Clinical Symptoms, N (%):   - Shortness of Breath - Hemoptysis - Runny Nose - Sore throat - Chills - Lethargy - Vomiting - Diarrhea - Myalgia - Arthralgia - Abdominal Pain - Headache | - 21 (43.8) - 3 (6.3) - 18 (37.5) - 9 (18.8) - 12 (25.0) - 12 (25.0) - 8 (16.7) - 1 (2.1) - 3 (6.3) - 3 (6.3) - 4 (7.3) - 13 (27.1) | - 156 (41.9) - 6 (1.6) - 128 (34.4) - 60 (16.1) - 58 (15.6) - 119 (32.0) - 46 (12.4) - 19 (5.1) - 24 (6.5) - 33 (8.9) - 60 (16.1) - 89 (23.9) | - 1.077 - 4.067 - 1.144 - 1.200 - 1.805 - 0.709 - 1.417 - 0.395 - 0.967 - 0.685 - 0.473 - 1.181 | - 0.587 – 1.975 - 0.983 – 16.825 - 0.614 – 2.131 - 0.552 – 2.606 - 0.886 – 3.674 - 0.356 – 1.411 - 0.625 – 3.216 - 0.052 – 3.021 - 0.280 – 3.340 - 0.202 – 2.325 - 0.164 – 1.365 - 0.599 – 2.331 | - 0.811 - 0.053 - 0.672 - 0.645 - 0.104 - 0.327 - 0.404 - 0.371 - 0.957 - 0.544 - 0.166 - 0.631 | | 2.716 |
| Laboratory Markers, mean ± SD   - Hemoglobin (g/dL) - Leucocyte (/µl) - Platelet (/µl) - Granulocyte (%) - Lymphocyte (%) | 13.0 ± 1.9   - 12.2 ± 6.6 - 243.4 ± 107.3 - 74.4 ± 14.8 - 17.0 ± 13.1 | 12.1 ± 2.2   - 11.3 ± 7.4 - 239.3 ± 134.3 - 69.9 ± 16.5 - 21.4 ± 14.4 | 1.235   - 1.016 - 1.000 - 1.019 - 0.974 | 1.057 – 1.443   - 0.977 – 1.056 - 0.998 – 1.003 - 0.996 – 1.044 - 0.947 – 1.002 | 0.008   - 0.431 - 0.839 - 0.109 - 0.069 | | - 1.099 - 0.999 |

Supplement 3: Univariate and multivariate logistic regression results are shown for SARI patients that had influenza vs SARI patients without influenza. Associations between characteristics (demographic, clinic, and laboratory) and influenza infection were determined with univariate binary logistic regression analysis. Due to the limited number of influenza cases relative to the number of candidate variables, a multivariate logistic regression with LASSO penalty was fit as an exploratory variable selection exercise. While we report the odds ratio output of the LASSO analysis, the values should be interpreted cautiously, and the analysis should be primarily viewed as exploratory variable selection. Statistical analyses were performed using SPSS version 22 (IBM Corporation, Armonk, NY, USA) and R version 3.6.0.
